# The Consequences of Fillings of Different Metals Coming in Contact

**Published:** 1867-09

**Authors:** R. P. Nevill


### ARTICLE III.

*The consequences of Fillings of different Metals coming in Contact.* By R. P. NEVILL, D.D.S.

IN these palmy days of professional progress, when all who desire may read, and learn ; when our libraries may be filled with text-books and periodical literature which will compare favorably with that of the medical or any other profession, it is very surprising to see men, who profess to be first class operators, and superior to the majority of dental practitioners, (in their own conceit;) men who hold enviable positions in the confidence of the public, performing operations wholly contrary to the most simple teachings of common sense.

Some months ago I was requested to visit a lady at her residence for the purpose of extracting a tooth. She had been confined to her room for some time, and complained of pain in the right side of her face, somewhat similar to neuralgia. She said it was not exactly like tooth-ache, but that it annoyed her day and night, and that she wished the first superior right molar removed, as she referred all the trouble to this tooth. Upon examining the tooth I found it had four fillings in it ; two of gold in the grinding surface, one of tin in the posterior approximal, and one of amalgam in the anterior approximal surface all of which seemed to be doing well. She informed me that the latter cavity had been filled with gold, which came out, and that her dentist had replaced it with amalgam about three weeks previous to the time I was called to see her.

Prior to this time she had experienced no trouble with her teeth, and none of the cavities were very sensitive when the fillings were inserted. The gum was healthy, except that it seemed to be somewhat redder than natural and slightly turgid. I did not feel that it would be proper to remove this tooth, as I saw no reason why it should cause all this trouble. I therefore requested her to have her medical advisor treat her for neuralgia. The next morning she sent for me again, her family physician being present, who stated that he did not think the affection was neuralgia, as it yielded to neither opiates nor quinine, and that he thought the tooth should be extracted immediately. I told him I thought differently, but that I would perform the operation if the patient determined to have the tooth removed.

The physician administered chloroform and the tooth was extracted. I then examined the tooth to ascertain the cause of the trouble, and found that the fangs were healthy. I removed the amalgam filling and discovered that it was in direct contact with the upper portion of one of the gold fillings ; none of the cavities approaching very near to the pulp. I concluded that galvanic action brought about by the two fillings of different metals, coming in contact, was the cause of the whole trouble, and that the tooth might have been saved by removing the amalgam filling and replacing it with gold. This case opened my eyes and I determined to be more cautious in future.

Another case, quite similar to the above, the symptoms being almost identical, presented itself some six months later. A lady called at my office to have a tooth extracted, complaining of pain in the first superior left molar and second bicuspid teeth, but could not tell which of these she wished extracted. The molar had a filling in the anterior approximal surface, and the bicuspid one in the posterior approximal surface, both being very much discolored; she was under the impression that they were both of tin. The space made by separating the teeth had closed up, so that the two fillings were in contact, and the bicuspid was a little loose. The teeth appeared healthy, and the fillings were apparently doing well. No decay was visible around their margins, and I refused to extract the teeth; she then insisted on having the fillings removed, which I also refused to do. Her teeth on that side needed scaling very badly, and in the lower jaw on the same side some were very much decayed. I removed the tartar and extracted the teeth which were too badly decayed to be restored to health. These operations were of no decided benefit, and she returned the next day determined to have one of the teeth referred to extracted.

On examining the fillings with an excavator I found that one was composed of amalgam and the other of tin. I stated to her that the fillings were not both of tin, and after a few moments reflection she remembered that the *dentist* had informed her that the teeth could not be filled with anything but some plastic material. With a file I made a free V shape separation, from which she experienced immediate relief, and before leaving the office informed me that she was entirely free from pain. She has had no trouble with her teeth since, and should this be the means of giving the least ray of light to guide the inexperienced, my object shall have been fully accomplished.

